# Dietary quality index and the risk of breast cancer: a case-control study

**DOI:** 10.1186/s12905-023-02588-6

**Published:** 2023-09-01

**Authors:** Fatemeh Pourhabibi-Zarandi, Masoud Amini Kahrizsangi, Sevda Eskandarzadeh, Fatemeh Mansouri, Mohebat Vali, Saba Jalali, Zeinab Heidari, Zainab Shateri, Mehran Nouri, Bahram Rashidkhani

**Affiliations:** 1 Department of Community Nutrition, School of Nutrition and Food Sciences, Shiraz University of Medical Sciences, Shiraz, Iran; 2 Student Research Committee, Shiraz University of Medical Sciences, Shiraz, Iran; 3 Department of Community Nutrition, School of Nutrition and Food Sciences, Isfahan University of Medical Sciences, Isfahan, Iran; 4 Department of Nutrition, School of Public Health, Iran University of Medical Sciences, Tehran, Iran; 5 Department of Clinical Nutrition, School of Nutrition and Food Sciences, Shiraz University of Medical Sciences, Shiraz, Iran; 6 Human Nutrition, Faculty of Land and Food Systems, University of British Columbia, Vancouver, Canada; 7 Student Research Committee, Ahvaz Jundishapur University of Medical Sciences, Ahvaz, Iran; 8 Department of Nutrition and Biochemistry, School of Medicine, Ilam University of Medical Sciences, Ilam, Iran; 9 Health Policy Research Center, Institute of Health, Shiraz University of Medical Sciences, Shiraz, Iran; 10 Department of Community Nutrition, Faculty of Nutrition and Food Technology, National Nutrition and Food Technology Research Institute, Shahid Beheshti University of Medical Sciences, Tehran, Iran

**Keywords:** Diet quality index, Diet quality index-international, Breast cancer, Breast neoplasms, Iranian

## Abstract

**Background:**

Diet quality is a significant determinant in the etiology of breast cancer (BrCa), but further studies are required to explore this relationship. Therefore, we tried to assess if diet quality, assessed using the Diet Quality Index-International (DQI-I), was related to BrCa among the Iranian population.

**Methods:**

In the present case-control research, 134 women with a recent diagnosis of BrCa and 267 without BrCa were selected as case and control groups. Individual food intake data from a food frequency questionnaire was used to compute DQI-I. Also, the multivariable logistic regression models were utilized to evaluate the association between DQI-I and BrCa odds .

**Results:**

We found a significant association between the last tertile of DQI-I and BrCa odds in the fully adjusted model (odds ratio (OR) = 0.30; 95% confidence interval (CI): 0.15–0.56). The subgroup analysis based on menopausal status also showed a significant decrease in BrCa odds in pre-and post-menopausal women (pre-menopausal: OR = 0.27; 95% CI: 0.10–0.70 – post-menopausal status: OR = 0.35; 95% CI: 0.13–0.92).

**Conclusions:**

Our findings indicated that a higher DQI-I score was related to a lower chance of BrCa. According to our research, a healthy diet pattern is crucial for BrCa prevention.

**Supplementary Information:**

The online version contains supplementary material available at 10.1186/s12905-023-02588-6.

## Introduction

The most prevalent cancer in women is breast cancer (BrCa), which has a significant mortality and morbidity rate in this group [1]. Data from the International Agency for Research on Cancer (IARC) shows 2.3 million new BrCa cases from 185 countries in 2018 [2]. The reports indicated 16,967 new BrCa cases and 4,810 deaths related to the disease in 2020 in Iran [3]. Also, based on Iran’s cancer registry data from 2008 to 2016, it is predicted that the incidence of BrCa in women will increase by 63% by 2025 [4].

Diet is one of the modifiable factors related to most cancer types, consisting of BrCa [5]. According to several studies, some foods and nutrients are considered protective or potential risk factors for BrCa. High carbohydrates, red and processed meats, and saturated fats are related to a higher progression and risk of BrCa [6–8]. In contrast, diets rich in fiber, minerals, omega-3 polyunsaturated fatty acids (PUFAs), antioxidants, fruits, vegetables, and vitamins have protective effects [9]. Although specific dietary factors have been shown to affect BrCa [5, 9–11], dietary patterns demonstrating the possible interactions between nutrients and foods can better evaluate the relationship between diet and the risk of BrCa [12, 13].

The diet quality index-international (DQI-I) is one of the dietary indices based on international dietary guidelines to investigate nutritional balance (0–10 score), moderation (0–30 score), variety (0–20 score), and adequacy (0–40 score), and the range of total index score is from 0 to 100, where greater scores demonstrate better diet quality [14, 15]. Recently, studies have indicated an inverse association between the DQI-I and risk factors for cardiovascular disease and cancer mortality [16, 17].

Considering the high prevalence of BrCa in Iran and the type of dietary patterns associated with higher consumption of saturated fats, refined grains, and the high percentage intake of energy from carbohydrates in the Middle East population, more studies on the association between BrCa and diet quality are necessary [3, 18]. The present research is one of the first to examine the association between diet quality and BrCa in a case-control design in Iranian women.

## Methods

### Study design

In the present case-control study, 136 women aged above 30 years with a recent BrCa diagnosis (less than six months) were recruited as a case group. For the control group, we recruited 272 women with non-neoplastic disorders and no long-term diet change who were not alcohol abusers. Based on a previous study by Ching et al., the study sample size was calculated (odds ratio (OR) = 0.47, α = 0.05, and β = 0.20) [19]. Patients with acute and non-neoplastic diseases hospitalized in the same hospital as the case group were selected from other hospital departments as a control group. The control group conditions were traumas and orthopedic disease, problems of the skin, nose, eyes or ear, and acute surgical conditions. We recruited all the participants from two Shohadaye Tajrish and Imam Hossein hospitals in Tehran (both case and control groups were selected from these hospitals). The study was conducted between September 2015 and February 2016. The number of people who withdrew from participating in the study at the same interview stage was less than 8%. In the final analysis, seven participants (five controls and two cases) were excluded because their caloric intake was out of three standard deviations (SDs) from the mean population calorie intake. Also, women with special diets or hormone replacement therapy or who were pregnant and lactating were excluded from the study. Ultimately, we conducted the final analysis on 134 cases and 267 controls.

This study was approved by the National Nutrition and Food Technology Research Institute of Iran, and informed consent was taken from all the participants.

### Data collection

Trained dietitians administered all measurements and questionnaires in the same interview. Also, a validated questionnaire was used to evaluate physical activity, and the results were reported as the metabolic equivalent of task-hours per day (MET-h/d) [20]. A non-stretchable tape meter fixed to a wall was used to measure their height (with an accuracy of 0.5 cm), and a digital scale with 0.1 kg accuracy was used to measure participants’ weight with minimum clothes and without shoes. Body mass index (BMI) was computed by dividing weight by the square of height (kg/m^2^). We also utilized a checklist to gather participants’ clinical information, lifestyle, and socio-demographic-economic including disease history (yes/no), bra wearing during the day and night (yes/no), age (year), marriage time (year), menopausal status (pre-and post-menopause), abortion history (yes/no), smoking history (yes/no), breastfeeding time (month), and taking medications and supplements (yes/no).

### Dietary intake assessment

To assess participants’ food intake one year before diagnosis in the case group or hospitalization for the control group, a valid and reliable semi-quantitative food frequency questionnaire (FFQ) (168 items) was applied [21]. Participants were requested to indicate the frequency (daily, weekly, monthly, or yearly) of consuming each food item. Their consumption was then converted to frequency per day, and using the handbook for household measures; intake frequencies were converted to grams per day [22]. The composition table of the United States Department of Agriculture (USDA) food [23] was utilized to compute most foods’ calorie and nutrient content. We used the composition table of Iranian foods for the traditional Iranian foods that were not in the USDA database [24].

### Diet quality assessment

We used DQI-I, consisting of four major dietary indicators, for diet quality assessment. The dietary components of DQI are as follows:

1. Food variety with two components including various types of food groups (legumes and legumes products, eggs, meats and products of meat, vegetables, fruits, grains, milk, and dairy products) and the types of protein sources within the group (legumes and their products, milk, meats, eggs), which has a score between 0 and 20.
2. Adequacy of foods used to assess protein, grains, fruits, vegetables, fiber, calcium, vitamin C, and ferric scored from 0 to 40 points.
3. Moderation of diet (cholesterol, saturated fat, total fat, empty calorie foods, and sodium), with a score between 0 and 30 points.
4. Diet balance (ratio of macronutrients and fatty acids), with a score of 0 to 10. The sum of the four above components is equal to the total score of DQI-I, which ranges from 0 to 100 (0 = the lowest and 100 = the highest dietary quality) [25].

### Statistical analysis

We used the chi-square test for categorical variables and Mann-Whitney or independent samples T-test for continuous variables to ascertain the general characteristics difference between the case and control groups. Values are percentage and median (25^th^, 75^th^ percentiles) or mean ± SD for categorical and continuous variables, respectively. Based on participants’ scores on DQI-I, we assigned them to tertiles. Analysis of variance (ANOVA) or Kruskal-Wallis were used to assess the nutrient intake across the tertiles of DQI-I. To evaluate the association between DQI-I and the odds of BrCa, the multivariable logistic regression models across the tertiles were applied. Variables like age, BMI, family history of cancer, family history of BrCa, energy, physical activity, taking vitamin D supplements, and menopausal status were adjusted to eliminate confounding effects (enter method). To carry out statistical analyses, SPSS (version 26.0, IBM Co., Chicago, IL) was used in the present study. We considered a p-value less than 0.05 as the significance level.

## Results

As shown in Table 1, age, abortion history, family history of cancer, adequacy score, moderation score, DQI-I total score, wearing a bra during the day, and taking vitamin D supplements significantly differed between the two groups (P<0.05).

**Table 1:** The baseline characteristics of the study population

Variables	Cases (134)	Controls (267)	P-value
Age (year) ^1^	49.5 ± 10.7	47.1 ± 10.1	**0.030**
Marriage age (year) ^2^	19.0 (16.0–22.0)	18.0 (16.0–20.0)	0.072
Age at first pregnancy (year) ^2^	20.0 (17.0–25.0)	20.0 (17.0–22.0)	0.053
Breastfeeding time (month) ^2^	39.0 (20.0–60.0)	48.0 (24.0–70.0)	0.162
BMI (kg/m^2^) ^2^	29.6 (25.9–33.3)	28.5 (25.4–31.6)	0.129
Physical activity (MET-h/day) ^2^	32.1 (29.1–35.3)	31.4 (29.1–34.9)	0.700
Variety score ^2^	20.0 (20.0–20.0)	20.0 (20.0–20.0)	0.230
Adequacy score ^2^	38.0 (36.0–39.0)	38.0 (37.0–39.0)	**0.009**
Moderation score ^2^	6.0 (3.0–9.0)	6.0 (6.0–9.0)	**0.007**
Overall balance score ^2^	5.0 (4.0–6.0)	5.0 (4.0–6.0)	0.668
DQI-I total score ^2^	67.6 (64.6–69.6)	69.0 (66.0–73.0)	**<0.001**
Energy (kcal/day) ^2^	2417.6 (2079.8-2956.7)	2549.6 (2149.9-3204.5)	0.072
Fiber (g/day) ^2^	33.5 (26.7–42.8)	35.4 (26.9–48.2)	0.175
Menopausal status, % ^3^ Pre-menopause Post-menopause	61 (45.9) 73 (54.1)	152 (57.2) 115 (42.8)	0.085
Smoking, no, % ^3^	130 (97.0)	258 (96.6)	1.000
Abortion history, no, % ^3^	81 (60.9)	190 (71.3)	**0.041**
Family history of breast cancer, no, % ^3^	123 (91.7)	255 (95.5)	0.171
Family history of cancer, no, % ^3^	93 (69.9)	211 (79.2)	**0.047**
Wearing a bra during the day, no, % ^3^	12 (9.0)	49 (18.6)	**0.012**
Wearing a bra at night, no, % ^3^	28 (21.1)	77 (28.8)	0.116
Vitamin D supplement, no, % ^3^	114 (85.0)	202 (75.8)	**0.037**
Omega-3, no, % ^3^	126 (94.0)	236 (88.3)	0.076
Herbal drugs, no, % ^3^	109 (81.2)	195 (73.1)	0.083

BMI: body mass index, MET: metabolic equivalent of task, DQI-I: dietary quality index-international

Values are mean ± SD or median (25^th^, 75^th^ percentiles) for continuous and number (percent) for categorical variables

^1^ Using independent samples T-test for normal continuous variables

^2^ Using Mann-Whitney for abnormal continuous variables

^3^ Using chi-square test for categorical variables

According to Table 2, monounsaturated fatty acids (MUFAs) (P = 0.010), PUFAs (P = 0.022), fruits (P = 0.019), vegetables (P = 0.031), and low-fat dairy (P = 0.025) intakes were significantly different between the case and control groups (the intake between the tertiles of case and control are shown in Supplementary Tables 1 and 2).

**Table 2:** Food group consumption based on the case and control group

Variables	Case (n = 134)	Control (n = 267)	P-value
Carbohydrate (energy %) ^1^	53.8 ± 6.7	54.2 ± 7.0	0.525
Protein (energy %) ^1^	12.7 ± 2.0	13.0 ± 2.1	0.113
SFA (energy %) ^1^	11.2 ± 2.0	10.8 ± 2.3	0.147
MUFA (energy %) ^1^	12.8 ± 3.1	12.0 ± 2.9	**0.010**
PUFA (energy %) ^1^	8.5 ± 2.9	7.8 ± 2.8	**0.022**
Whole Grains (g/day) ^2^	70.6 (33.3-111.5)	66.6 (39.2-116.7)	0.350
Refined Grains (g/day) ^2^	275.9 (204.7-335.6)	282.9 (180.7-387.1)	0.397
Fruits (g/day) ^2^	426.9 (334.3-583.8)	496.6 (362.6-645.3)	**0.019**
Vegetables (g/day) ^2^	298.9 (220.7-472.9)	360.9 (268.9-463.3)	**0.031**
Red Meats (g/day) ^2^	19.2 (10.5–28.7)	19.4 (13.0-28.7)	0.472
Poultry (g/day) ^2^	54.8 (26.8–73.4)	54.8 (28.1–80.8)	0.708
Fishes (g/day) ^2^	7.7 (3.7–15.7)	9.4 (4.1–18.3)	0.136
Processed Meats (g/day) ^2^	1.6 (0.3-6.0)	1.3 (0.0-4.6)	0.182
Organ Meats (g/day) ^2^	3.6 (0.8–7.3)	2.4 (0.9–6.4)	0.116
Low-Fat Dairy (g/day) ^2^	560.6 (316.9-809.5)	666.7 (421.8–906.0)	**0.025**
High-Fat Dairy (g/day) ^2^	40.2 (12.6-232.1)	46.2 (12.6–236.0)	0.884
Legumes (g/day) ^2^	23.1 (14.1–38.6)	27.2 (15.3–44.1)	0.255
Nuts (g/day) ^2^	7.8 (3.1–15.0)	9.1 (4.2–16.1)	0.058
Snacks (g/day) ^2^	16.3 (8.1–38.0)	16.3 (5.4–44.1)	0.595
Sweets (g/day) ^2^	43.1 (25.8–70.7)	37.4 (23.7–56.9)	0.100
Liquid Oils (g/day) ^2^	17.4 (9.1–24.0)	15.0 (6.4–25.7)	0.725
Solid Oils (g/day) ^2^	14.6 (5.2–26.4)	10.7 (3.6–26.4)	0.087
Sugar-Sweetened Beverages (g/day) ^2^	16.3 (2.0-40.8)	8.1 (1.3–35.0)	0.079

DQI-I: dietary quality index-international, SFA: saturated fatty acids, MUFA: monounsaturated fatty acids, PUFA: polyunsaturated fatty acids, g: gram

Values are mean ± SD or median (25^th^, 75^th^ percentiles) for continuous

^1^ Using ANOVA for normal continuous variables

^2^ Using Kruskal-Wallis U test for abnormal continuous variables

As shown in Table 3, there was a significant negative relationship between DQI-I and BrCa odds in both Model 1 (OR = 0.29; 95% confidence interval (CI): 0.16–0.51) and Model 2 (OR = 0.30; 95% CI: 0.15–0.56).

**Table 3:** Association between DQI-I and breast cancer

Tertiles of Index	Case/Control	Model 1		Model 2	
		OR	95% CI	OR	95% CI
T_1_ (≤ 66.66)	55/78	1.00	Ref.	1.00	Ref.
T_2_ (66.67–70.33)	55/79	0.98	0.60–1.60	0.98	0.56–1.72
T_3_ (≥ 70.34)	24/110	**0.29**	**0.16–0.51**	**0.30**	**0.15–0.56**
P_trend_		**<0.001**		**<0.001**	

DQI-I: dietary quality index-international

Model 1: adjusted for age

Model 2: adjusted for age, marriage age, age at first pregnancy, breastfeeding time, BMI, family history of breast cancer, family history of cancer, energy, physical activity, smoking history, wearing a bra during the day and night, taking vitamin D supplements, and menopausal status

Obtained from logistic regression

These values are odds ratio (95% CIs).

Significant values are shown in bold.

The subgroup analysis based on menopausal status is shown in Table 4. As presented in the table, in the Model 1, we observed a significant reduction in the odds of BrCa in pre-and post-menopausal participants (pre-menopausal: OR = 0.21; 95% CI: 0.08–0.50 – post-menopausal status: OR = 0.35; 95% CI: 0.16–0.76). In addition, after adjusting for many potential confounders, this association remained significant (pre-menopausal: OR = 0.27; 95% CI: 0.10–0.70 – post-menopausal status: OR = 0.35; 95% CI: 0.13–0.92).

**Table 4:** Association between DQI-I and breast cancer by menopausal status

Tertiles of Index	Case/Control	Model 1			Model 2	
		OR	95% CI		OR	95% CI
**Pre-menopausal**						
T_1_ (≤ 66.66)	28/47	1.00	Ref.		1.00	Ref.
T_2_ (66.67–70.33)	25/39	1.07	0.54–2.13		1.23	0.54–2.78
T_3_ (≥ 70.34)	8/65	**0.21**	**0.08–0.50**		**0.27**	**0.10–0.70**
P_trend_		**0.001**			**0.012**	
**Post-menopausal**						
T_1_ (≤ 66.66)	27/29	1.00		Ref.	1.00	Ref.
T_2_ (66.67–70.33)	30/39	0.84		0.41–1.72	0.79	0.33–1.90
T_3_ (≥ 70.34)	15/46	**0.35**		**0.16–0.76**	**0.35**	**0.13–0.92**
P_trend_		**0.009**			**0.034**	

DQI-I: dietary quality index-international

Model 1: adjusted for age

Model 2: adjusted for age, marriage age, age at first pregnancy, breastfeeding time, BMI, family history of breast cancer, cancer family history, energy, physical activity, smoking history, wearing bra at day and night, and taking vitamin D supplements

Obtained from logistic regression

These values are odds ratio (95% CIs).

Significant values are shown in bold.

## Discussion

According to our findings, higher DQI-I scores were related to a lower odds of BrCa. Furthermore, based on the fully adjusted model, the association remained strengthened, which shows that this relationship is robust. Also, after splitting according to menopausal status, a decreased risk of BrCa was observed with an increase in DQI-I score in post-menopausal and pre-menopausal women, with a greater reduction in pre-menopausal women.

Our literature review reveals that the current research is among the first to assess an association between DQI-I and the odds of BrCa. The present study’s findings are consistent with previous studies assessing the possible association between DQI and cancer risks. Park et al., Vulcan et al., and Vargas et al. demonstrated that higher diet quality scores are associated with a reduced risk of colorectal cancer [26–28]. In another study, Wang et al. found that more adherence to high diet quality predicted a lower risk of nasopharyngeal carcinoma [29]. Sohouli et al. demonstrated no association between any particular DQI-I quartile and BrCa. However, the trend was substantial across all quartile categories [30]. However, our results showed that people in the highest tertile compared to the lowest tertile of DQI-I scores had a significantly lower odds of BrCa. Inconsistent with our results, Godoy et al. reported no association between the DQI and its components with BrCa risk [31].

When women were stratified by menopausal status, higher DQI-I scores were related to a decreased risk of BrCa with a more decrease in pre-menopausal women. In fact, the findings showed that in higher DQI-I, the odds of BrCa in pre-and post-menopausal women decreases by 73% and 65%, respectively. Due to the limitation of studies on the relationship between DQI-I score and BrCa, the results of other dietary quality indices were reviewed. A prospective cohort study observed an inverse association between higher diet quality scores (alternate Mediterranean diet, DASH, and alternative healthy eating index–2010) and BrCa incidence in post-menopausal women. However, no association was observed between diet quality indices and BrCa incidence in pre-menopausal women [32]. In a systematic review, the association between diet quality scores and the risk of post-menopausal BrCa was inverse [33]. A case-control study revealed that pre-menopausal women had a decreased risk of BrCa in the highest quartile of the healthy pattern. In contrast, post-menopausal women’s diet patterns did not differ significantly from each other [34].

Menopause is not a cause of BrCa, but with ageing, the risk of developing women-related cancers such as ovarian, uterine, and breast increases [35]. As an unmodifiable risk factor, age leads to cellular and molecular changes in normal breast tissue that can lead to malignant transformation [36]. Two-thirds of newly diagnosed women with BrCa are post-menopausal, age 55 and older [37]. In the current study, the case group was older than the control group.

DQI-I is a diet quality indicator that assesses four diet quality components: diversity, adequacy, moderation, and balance [14]. A higher DQI-I score is related to a healthier dietary pattern containing bioactive substances and various vitamins and minerals that can reduce the risk of BrCa [29]. In this study, the higher total DQI-I score and the higher score of the adequacy and moderation components in the control group compared to the cases were consistent with our expectations.

Vitamin D plays a significant role in differentiation and subsequently influences the function and development of the mammary gland [38]. Vitamin D deficiency is related to the pathogenesis of different types of cancers like BrCa [39]. Although the results of studies on the dose of vitamin D and the risk of BrCa are conflicting, according to a dose-response meta-analysis, an increasing inverse relationship was observed above the threshold of 27 ng/mL in post-menopausal women but not in pre-menopausal. This effect reached a steady state in doses above 35 ng/mL [40]. In a randomized clinical trial, supplementation with calcium and vitamin D (400 IU vitamin D with 1 gr Ca /day) in post-menopausal women caused a significant decrease in the risk of both breast and invasive breast cancers by 14-20% [41]. The results of our study also revealed that the case group used vitamin D supplements less frequently than the control group. Due to the nature of the study design, there was no control over the subjects’ vitamin D intake.

Using logistic regression models with probable confounder adjustments is one of the study’s strengths. In addition, the validated FFQ represents the most types of foods that our study subjects consumed. To our knowledge, this is one of the first studies to investigate the association between DQI-I and BrCa. Nevertheless, there are several limitations that should be noted. The main limitation of the current study is its retrospective and case-control design, which makes it impossible to determine causality. Because of using FFQ, recall bias is probable. Also, using self-report methods can lead to over-or under-reporting. However, to minimize these problems, skilled and trained staff were employed to conduct the interviews.

## Conclusions

In conclusion, the present study illustrated that a higher DQI-I score was related to a lower chance of BrCa. Also, a decreased odds of BrCa was found with an increase in DQI-I score in both post-and pre-menopausal women, with a higher decrease in pre-menopausal women. According to our research, a healthy diet pattern is crucial for BrCa prevention. Further investigations with different designs, especially prospective cohort studies, are required to support these results.

### Electronic supplementary material

Below is the link to the electronic supplementary material.

Additional File 1: Table 1 and 2

## Data Availability

The datasets used and/or analyzed during the current study are available from the corresponding author on reasonable request.
