# Supplementary material for: Dietary quality index and the risk of breast cancer: a case-control study
Source: BMC Womens Health. 2023 Sep 1;23:469. doi: 10.1186/s12905-023-02588-6 (PMC10474712; doi:10.1186/s12905-023-02588-6)
Supplement: Supplementary file 1 — Additional File 1: Table 1 and 2 [file 12905_2023_2588_MOESM1_ESM.docx]

**Supplementary Table 1.** Food group consumption based on the DQI-I tertiles in the case group.

| **Variables** | **T_1_ (n=55)** | **T_2_ (n=55)** | **T_3_ (n=24)** | **P-value** |
| --- | --- | --- | --- | --- |
| Carbohydrate (energy %) ^1^ | 51.7 ± 6.6 | 54.2 ± 6.7 | 57.7 ± 4.6 | **0.001** |
| Protein (energy %) ^1^ | 12.9 ± 2.1 | 12.4 ± 2.0 | 12.6 ± 1.8 | 0.525 |
| SFA (energy %) ^1^ | 12.2 ± 2.1 | 11.0 ± 1.3 | 9.0 ± 1.4 | **˂0.001** |
| MUFA (energy %) ^1^ | 13.3 ± 3.2 | 13.1 ± 3.2 | 11.2 ± 1.9 | **0.024** |
| PUFA (energy %) ^1^ | 8.3 ± 2.9 | 8.9 ± 3.3 | 7.8 ± 1.9 | 0.356 |
| Whole Grains (g/day) ^2^ | 52.7 (33.6-97.8) | 77.2 (27.4-111.1) | 82.8 (48.5-132.3) | 0.292 |
| Refined Grains (g/day) ^2^ | 265.8 (215.7-335.5) | 277.9 (203.5-328.1) | 290.7 (168.5-363.2) | 0.923 |
| Fruits (g/day) ^2^ | 386.3 (311.5-481.8) | 426.9 (344.6-570.9) | 574.6 (467.3-653.2) | **0.002** |
| Vegetables (g/day) ^2^ | 268.1 (191.3-385.9) | 314.6 (244.1-533.8) | 416.7 (296.5-545.9) | **0.008** |
| Red Meats (g/day) ^2^ | 20.0 (10.0-27.4) | 17.9 (10.3-29.0) | 18.9 (12.5-28.3) | 0.839 |
| Poultry (g/day) ^2^ | 54.8 (27.4-73.1) | 48.7 (25.3-76.4) | 36.6 (26.2-81.8) | 0.841 |
| Fishes (g/day) ^2^ | 8.5 (3.9-15.9) | 6.1 (3.6-12.5) | 11.0 (3.8-17.2) | 0.443 |
| Processed Meats (g/day) ^2^ | 2.1 (0.5-6.1) | 1.4 (0.0-5.8) | 1.0 (0.3-6.0) | 0.345 |
| Organ Meats (g/day) ^2^ | 4.4 (2.3-9.6) | 3.1 (0.9-6.0) | 0.9 (0.6-5.2) | **0.006** |
| Low-Fat Dairy (g/day) ^2^ | 635.1 (384.8-830.3) | 560.6 (258.7-787.1) | 412.8 (236.2-675.0) | 0.129 |
| High-Fat Dairy (g/day) ^2^ | 74.8 (13.3-241.6) | 37.1 (14.4-233.0) | 33.6 (9.7-166.5) | 0.405 |
| Legumes (g/day) ^2^ | 22.5 (13.5-32.8) | 23.4 (14.1-42.9) | 26.4 (16.9-45.4) | 0.259 |
| Nuts (g/day) ^2^ | 6.5 (2.7-9.9) | 9.2 (3.6-14.9) | 9.9 (2.7-17.9) | 0.113 |
| Snacks (g/day) ^2^ | 16.3 (7.7-38.0) | 16.9 (10.8-38.5) | 16.3 (4.7-52.1) | 0.714 |
| Sweets (g/day) ^2^ | 40.1 (28.1-65.0) | 44.2 (22.3-71.3) | 52.5 (27.-80.8) | 0.497 |
| Liquid Oils (g/day) ^2^ | 12.4 (5.8-22.5) | 18.0 (11.9-28.2) | 18.0 (7.5-26.8) | 0.346 |
| Solid Oils (g/day) ^2^ | 19.0 (8.2-31.3) | 11.9 (4.5-25.7) | 10.2 (1.8-22.6) | 0.060 |
| Sugar-Sweetened Beverages (g/day) ^2^ | 16.3 (2.2-35.0) | 8.1 (3.7-70.0) | 20.4 (1.3-35.0) | 0.930 |

DQI-I: dietary quality index-international, SFA: saturated fatty acids, MUFA: monounsaturated fatty acids, PUFA: polyunsaturated fatty acids, g: gram.

Values are mean ± SD or median (25^th^, 75^th^ percentiles) for continuous.

^1^ Using ANOVA for normal continuous variables.

^2^ Using Kruskal-Wallis U test for abnormal continuous variables.

**Supplementary Table 2.** Food group consumption based on the DQI-I tertiles based on control group.

| **Variables** | **T_1_ (n=78)** | **T_2_ (n=79)** | **T_3_ (n=100)** | **P-value** |
| --- | --- | --- | --- | --- |
| Carbohydrate (energy %) ^1^ | 50.2 ± 5.6 | 52.9 ± 6.5 | 57.9 ± 6.4 | **˂0.001** |
| Protein (energy %) ^1^ | 13.2 ± 2.3 | 12.7 ± 1.9 | 13.1 ± 2.1 | 0.344 |
| SFA (energy %) ^1^ | 12.8 ± 2.1 | 11.3 ± 1.5 | 9.0 ± 1.6 | **˂0.001** |
| MUFA (energy %) ^1^ | 13.2 ± 2.5 | 12.5 ± 3.2 | 10.8 ± 2.6 | **˂0.001** |
| PUFA (energy %) ^1^ | 8.1 ± 2.6 | 8.1 ± 3.3 | 7.3 ± 2.6 | 0.072 |
| Whole Grains (g/day) ^2^ | 56.9 (33.1-89.3) | 65.8 (36.9-108.1) | 78.5 (47.7-173.9) | **0.014** |
| Refined Grains (g/day) ^2^ | 229.8 (150.6-344.0) | 264.4 (180.7-328.3) | 314.6 (239.3-524.4) | **˂0.001** |
| Fruits (g/day) ^2^ | 432.0 (331.2-545.9) | 489.7 (396.5-651.6) | 476.7 (393.2-679.6) | **0.038** |
| Vegetables (g/day) ^2^ | 342.6 (267.3-438.6) | 343.7 (246.6-450.3) | 398.8 (290.9-498.6) | 0.081 |
| Red Meats (g/day) ^2^ | 20.7 (15.0-29.7) | 20.3 (12.9-28.9) | 17.2 (12.9-25.3) | 0.121 |
| Poultry (g/day) ^2^ | 54.8 (27.4-73.1) | 54.6 (28.3-82.3) | 54.8 (27.4-79.3) | 0.915 |
| Fishes (g/day) ^2^ | 10.7 (5.3-18.3) | 7.4 (3.6-16.5) | 9.8 (4.9-18.6) | 0.266 |
| Processed Meats (g/day) ^2^ | 1.9 (0.4-4.5) | 1.5 (0.0-4.4) | 1.0 (0.0-4.8) | 0.397 |
| Organ Meats (g/day) ^2^ | 2.9 (1.3-8.2) | 2.1 (0.5-4.5) | 2.7 (0.8-6.8) | 0.140 |
| Low-Fat Dairy (g/day) ^2^ | 661.2 (430.6-833.4) | 701.0 (526.8-936.7) | 600.1 (365.3-886.2) | 0.168 |
| High-Fat Dairy (g/day) ^2^ | 50.3 (15.0-307.1) | 66.3 (10.0-238.5) | 34.6 (10.1-141.5) | 0.100 |
| Legumes (g/day) ^2^ | 25.3 (14.5-38.6) | 26.9 (15.5-40.6) | 28.3 (15.2-49.7) | 0.478 |
| Nuts (g/day) ^2^ | 8.3 (4.2-16.7) | 8.6 (4.1-12.9) | 10.1 (4.8-18.2) | 0.357 |
| Snacks (g/day) ^2^ | 16.6 (5.8-44.1) | 20.1 (4.6-57.5) | 13.3 (5.5-34.3) | 0.436 |
| Sweets (g/day) ^2^ | 34.9 (22.1-52.3) | 41.1 (26.1-66.7) | 36.5 (25.5-56.0) | 0.199 |
| Liquid Oils (g/day) ^2^ | 13.7 (9.0-28.9) | 12.0 (5.1-25.6) | 18.0 (9.0-25.7) | 0.188 |
| Solid Oils (g/day) ^2^ | 14.7 (7.2-34.9) | 16.8 (4.1-33.3) | 7.1 (1.6-14.8) | **˂0.001** |
| Sugar- Sweetened Beverages (g/day) ^2^ | 8.1 (3.3-35.0) | 8.1 (1.3-35.0) | 8.1 (1.3-35.0) | 0.776 |

DQI-I: dietary quality index-international, SFA: saturated fatty acids, MUFA: monounsaturated fatty acids, PUFA: polyunsaturated fatty acids, g: gram.

Values are mean ± SD or median (25^th^, 75^th^ percentiles) for continuous.

^1^ Using ANOVA for normal continuous variables.

^2^ Using Kruskal-Wallis U test for abnormal continuous variables.
